# Postoperative Complications in Humanitarian Paediatric Patients Undergoing Late Surgical Correction of Tetralogy of Fallot: A Multivariate Analysis

**DOI:** 10.3390/children12091111

**Published:** 2025-08-23

**Authors:** Vitor Mendes, Samuel Filliol, Tomasz Nalecz, Ana Abecasis, Telmo Pereira, Maria do Rosário Oliveira Martins, Julie Wacker, Tornike Sologashvili

**Affiliations:** 1 Division of Cardiovascular Surgery, Department of Surgery, Geneva University Hospitals (HUG), 1211 Geneva 14, Switzerland; samuel.filliol@hcuge.ch (S.F.); tomasz.nalecz@hug.ch (T.N.); tornike.sologashvili@hug.ch (T.S.); 2 Global Health and Tropical Medicine (GHTM), Associate Laboratory in Translation and Innovation Towards Global Health (LA-REAL), Instituto de Higiene e Medicina Tropical (IHMT), Universidade NOVA de Lisboa, 1349-008 Lisboa, Portugal; ana.abecasis@ihmt.unl.pt (A.A.); mrfom@ihmt.unl.pt (M.d.R.O.M.); 3 H&TRC—Health & Technology Research Center, Coimbra Health School, Polytechnic University of Coimbra, 3045-043 Coimbra, Portugal; telmo@estesc.ipc.pt; 4 Pediatric Cardiology Unit, Department of Women, Child and Adolescent Medicine, Geneva University Hospitals (HUG), University of Geneva, 1211 Geneva 14, Switzerland; julie.wacker@hug.ch

**Keywords:** Tetralogy of Fallot, humanitarian patients, late complete surgical repair, postoperative complications

## Abstract

**Background:** Tetralogy of Fallot is a congenital heart defect that requires early surgical correction. However, in developing countries, many patients undergo delayed treatment due to limited healthcare resources. This study aims to identify risk factors for postoperative complications in humanitarian patients undergoing late Tetralogy of Fallot repair, defined as surgery performed after 12 months of age. **Methods:** A retrospective analysis was conducted on 115 humanitarian paediatric patients with a median age of 1444 days (approximately 4 years) who underwent complete Tetralogy of Fallot correction. In this humanitarian programme, patients from developing nations underwent surgical repair at our tertiary referral centre in a high-resource country. Postoperative complications were monitored within the first 30 days after surgery. Two multivariable logistic regression models were used to analyse pre/perioperative (Model 1) and postoperative (Model 2) risk factors for complications. **Results:** Complications occurred in 24.3% of patients. No deaths were recorded. In Model 1, smaller pulmonary valve annulus (OR = 0.066; *p* < 0.01) and the use of right ventricle to pulmonary artery conduit (OR = 13.252; *p* < 0.01) were significantly associated with a higher risk of complications. In Model 2, prolonged invasive ventilation time (OR = 1.068; *p* < 0.01) and extended hospitalisation time (OR = 1.093; *p* = 0.04) were significantly associated with complications. **Conclusions:** Late surgical correction of Tetralogy of Fallot in humanitarian paediatric patients can be performed with low mortality but carries a significant risk of postoperative complications. The predictive models provide useful tools for proactive clinical monitoring, personalised management, and optimisation of hospital resources.

## 1. Background

Tetralogy of Fallot (TOF) is the most common cyanotic congenital heart disease, representing 3–5% of all congenital heart defects, with an incidence of 0.28–0.34 per 1000 live births [1]. Embryologically, TOF is characterised by an anterosuperior deviation of the conal septum, leading to pulmonary stenosis and a ventricular septal defect (VSD) [2]. The severity of the condition is directly related to the degree of pulmonary stenosis [3].

In untreated TOF patients, the mortality rate is notably high, with survival rates of 66% at one year of age and only 24% at ten years [4]. Complete surgical repair is widely considered the best therapeutic option. In specialised centres in developed countries, this procedure is recommended between 3 and 11 months of age [1]. This timing is intended to minimise the morbidity and mortality associated with TOF while optimising postoperative outcomes. In case of unfavourable preoperative factors, such as severe obstruction of right ventricular outflow tract (RVOT), low birth weight, or prematurity, a palliative procedure such as a systemic-to-pulmonary shunt or stenting of the RVOT may be necessary to delay complete repair [3].

In developed countries, mortality rates associated with total TOF repair have steadily decreased, despite the lowering of the age at surgery, with mortality rates frequently falling below 1% [1].

In developing countries, limited healthcare access and specialised expertise often result in delayed diagnosis and surgical correction. Most children with TOF in these regions do not have access to early palliative or corrective procedures and are, therefore, exposed to the prolonged consequences of hypoxaemia, including polycythaemia, cyanotic nephropathy, systolic and diastolic dysfunction, and impaired neurological development [1,3]. Furthermore, malnutrition, often associated with both the cardiac condition and unfavourable socioeconomic factors, increases perioperative risk [1]. All of these factors contribute to a higher incidence of postoperative complications.

Our centre, in collaboration with humanitarian organisations, provides surgical correction for many children from developing countries, including regions in Eastern Europe, North Africa, and sub-Saharan Africa. For the purpose of this study, “humanitarian patients” refers to children from underdeveloped countries who were referred to our centre by humanitarian organisations for surgical intervention. Currently, surgical repair with preservation of the native pulmonary valve annulus is encouraged, as it is associated with better long-term outcomes and a lower risk of pulmonary valve regurgitation [5,6].

This study aims to analyse the complications that occur within the first 30 days postoperatively in humanitarian paediatric patients who undergo surgical correction for TOF. The primary focus is to identify risk predictors associated with these complications, based on preoperative, perioperative, and postoperative parameters, in order to improve clinical outcomes and surgical planning.

## 2. Methods

### 2.1. Study Design and Patients

This single-centre retrospective observational study included paediatric humanitarian patients who were admitted for corrective surgery of TOF at Geneva University Hospitals (HUG) between January 2019 and May 2023.

All paediatric patients (aged < 18 years) referred to our centre by a humanitarian organisation with a confirmed diagnosis of TOF and who underwent complete surgical repair were included in the study. Patients who had undergone palliative cardiac interventions prior to the complete repair at our centre, along with those who had extracardiac malformations requiring surgical interventions during the same cardiopulmonary bypass (CPB) used for the cardiac surgery, were excluded.

Humanitarian patients were defined as those from underdeveloped countries, referred to our centre by a humanitarian organisation for surgical intervention.

Complete surgical repair of TOF involves the closure of the VSD with a patch and the relief of right ventricular outflow tract obstruction (RVOTO). Depending on the anatomy and the quality of the cardiac tissue, RVOTO relief can be categorised into three approaches: placement of an enlargement patch with preservation of the native pulmonary valve annulus (pulmonary valve-sparing repair (PV-SR)), placement of a transannular patch without preservation of the native pulmonary valve annulus (Transannular patch), and replacement of the RV-PA connection using a biological prosthesis (homograft or Contegra^®^) (RV-PA conduit). The primary strategy is to preserve the native annulus, with the other two approaches serving as alternatives when preservation of the functional native pulmonary valve is not feasible.

At our centre, the choice of surgical technique was determined intraoperatively following direct anatomical inspection. The surgical team prioritised preservation of the native pulmonary valve whenever technically possible. Factors influencing this decision included the absolute diameter and Z-score of the pulmonary valve annulus, the degree of annular and valvular dysplasia, and the feasibility of achieving adequate relief of RVOTO without disrupting the annulus. No fixed preoperative protocol dictated this choice beyond the intraoperative anatomical assessment.

Ethical clearance was obtained from the Regional Research Ethics Committee (CCER 2024-00407), with the requirement for informed consent waived due to the retrospective nature of the study. The study was conducted in line with the ethical principles outlined in the Declaration of Helsinki [7].

Demographic data (gender, age at the time of surgical correction, weight, height, body surface area, body mass index (BMI), country of origin, peripheral pulse oximetry, haemoglobin), preoperative data (maximal instantaneous RVOT gradient, presence and type of cardiac malformations associated with TOF), perioperative data (type of RVOTO relief approach, CPB time, aortic cross-clamp time), and postoperative data (invasive mechanical ventilation time, intensive care unit (ICU) length of stay, hospitalisation length of stay, complications within 30 days postoperative) were obtained from the clinical records of the selected patients.

Postoperative complications were categorised as described in the literature: death, need for extracorporeal membrane oxygenation (ECMO) support, neurological deficit, delayed chest closure, surgical reintervention within 30 days, acute renal failure, prolonged cardiovascular pharmacological support, chylothorax, respiratory infection requiring re-intubation, and arrhythmia. This categorisation is in line with similar studies, which used comparable criteria for postoperative outcomes [3,8].

Acute renal failure was defined according to paediatric KDIGO criteria (increase in serum creatinine by ≥0.3 mg/dL within 48 h or ≥1.5 times the baseline value within 7 days) [9].

Neurological deficits were defined as new-onset postoperative neurological symptoms documented in discharge summary, including transient paresis, altered level of consciousness, postoperative seizures. No cases of stroke were recorded.

For the purpose of statistical analysis, all postoperative complications—regardless of severity—were grouped into a single category to increase the study’s statistical power.

### 2.2. Statistical Analysis

After data collection and summarisation, statistical analysis was conducted using the “Statistical Package for the Social Sciences” (SPSS) version 27.

To characterise the sample, simple descriptive statistics were performed, including the calculation of central tendency (mean, median) and dispersion measures (standard deviation, interquartile range), as well as absolute and relative frequencies, as appropriate.

For the comparison of qualitative variables, Pearson’s chi-square test (χ^2^) or Fisher’s exact test was used whenever the conditions for the applicability of the chi-square test were not met. The degree of association between qualitative variables across the two groups was measured using Cramer’s V. Interpretation of Cramer’s V was as follows: values below 0.1 indicated a negligible association, values between 0.1 and 0.3 indicated a weak association, values between 0.3 and 0.5 indicated a moderate association, and values above 0.5 indicated a strong association [10].

The Shapiro–Wilk (SW) test was used to assess the normality of the distribution of quantitative variables. A normal distribution indicated the use of parametric statistical tests. In the absence of normality, non-parametric tests were applied.

For the comparison of quantitative variables between two groups, the independent samples *t*-test or the Mann–Whitney test was used when the assumptions of normality and homogeneity of variances (Levene’s test) were not met.

To quantify the size of the effect for significant differences between groups, Cohen’s d was calculated for the independent samples *t*-test, while the Pearson’s r (derived from the Z statistic) was used as the effect size measure for the Mann–Whitney test. Effect sizes were interpreted based on common benchmarks: for Cohen’s d, values of 0.2, 0.5, and 0.8 indicated small, medium, and large effects, respectively [10,11]. For Pearson’s r values of 0.1, 0.3, and 0.5 indicated small, medium, and large effects, respectively [11].

To better describe the association between the variables “complications” and a set of explanatory variables, a univariate binary logistic regression was performed, calculating the Odds Ratios (OR) and corresponding 95% Confidence Intervals (95% CI) for each variable, and testing the statistical significance of each coefficient to determine if it was significantly different from zero.

Subsequently, two separate multivariate binary logistic regression models were built to identify independent predictors of “complications”. Model 1 included preoperative and perioperative variables, while Model 2 included postoperative variables. Variables showing a *p*-value <0.05 in univariate analyses and/or deemed clinically relevant by the study team were considered as candidates for inclusion in the multivariate logistic regression models.

Multicollinearity among the explanatory variables was assessed using Tolerance and Variance Inflation Factor (VIF) tests, selecting only variables with a tolerance > 0.1 and VIF < 10.

The variable inclusion method used was “Forward Stepwise”, based on clinical relevance, to identify significant independent variables.

Given the potential to increase model complexity, interactions between exploratory variables (factors) were not added. The performance of the final model was evaluated using the -2 Log Likelihood, Nagelkerke R^2^, and the Hosmer–Lemeshow test.

The Receiver Operating Characteristic (ROC) curve analysis was applied to evaluate the predictive capacity of each model, with sensitivity and specificity calculated for each cutoff point to assess the model’s diagnostic accuracy.

The interpretation of statistical tests and the construction of ROC curves were conducted based on a significance level of α = 0.05 with 95% CI.

## 3. Results

A total of 115 paediatric patients who underwent corrective surgery for TOF were included. There were no recorded deaths among the individuals in the sample; however, 28 patients (24.3%) experienced complications within the first 30 days post-surgery. The complications included the following: ECMO support in one patient (0.9%), transient neurological deficits in four patients (3.5%), delayed chest closure in four patients (3.5%), surgical reintervention (<30 days post-surgery) in eight patients (7.0%), acute renal failure without the need for dialysis in one patient (0.9%), prolonged cardiovascular pharmacological support (>24 h) in seven patients (6.1%), chylothorax in one patient (0.9%), respiratory infection requiring re-intubation in one patient (0.9%), and reversible arrhythmia after electrolyte correction in one patient (0.9%).

Baseline data, including demographic, physical, and laboratory characteristics, are reported in Table 1.

The majority of our patients were male (n = 69; 60%) with a median age of 1444 days. All individuals had a chronic cyanotic form of TOF with a median peripheral pulse oximetry of 80% [70.00–87.00] and elevated haemoglobin levels (17.21 ± 4.12 g/dL). In the comparative analysis, no significant differences were observed between the two groups in terms of demographic, physical, and laboratory characteristics.

Table 2 shows the additional cardiac malformations associated with TOF, as well as the anatomical and haemodynamic characteristics observed through transthoracic echocardiography.

The majority of the individuals in our sample had an interatrial shunt (n = 89;77.4%), and nearly half presented with a persistent ductus arteriosus (n = 51; 44.7%). However, when comparing the two groups under analysis, no significant differences were observed in the prevalence of cardiac malformations associated with TOF.

Upon examination of the echocardiographic measurements, we identified significant differences between the two groups, with RVOT hypoplasia being more pronounced in the group with complications. Specifically, the pulmonary valve annulus diameter (cm) showed a *p*-value of <0.01, with a large effect size (Cohen’s d = 0.81), suggesting a strong practical relevance of this measure in distinguishing patients with complications. The pulmonary annulus Z-score, although statistically significant (*p* < 0.01), showed a weaker correlation with complications (Pearson’s r = −0.26), indicating a modest association and suggesting limited practical relevance as a predictor of complications.

The perioperative characteristics of our cohort can be analysed in Table 3.

Most of the surgical interventions were performed electively (n = 87; 77%). Although a higher proportion of urgent procedures was observed in the group with complications (32.1% vs. 20.0%), this difference was not statistically significant (*p* = 0.20). An association was found between the RVOTO surgical technique and the presence of complications (*p* < 0.01). The Cramer’s V value of 0.35 indicates a moderate association. Regarding the median of CPB time, significant differences were observed between the groups (*p* < 0.01), revealing a longer time in the group with complications (87.5 [64.3–96.8]) min. However, the correlation between CPB time and complications was weak (Pearson’s r= −0.23), suggesting that, although the difference is statistically significant, the practical relevance of this finding is modest.

The analysis of postoperative parameters, as well as the echocardiographic measurements at 30 post-operative days, can be found in Table 4.

Significant differences were observed between the groups regarding the median duration of invasive ventilation after CPB weaning (*p* < 0.01), as well as the ICU time (*p* < 0.01) and hospitalisation time (*p* < 0.01), with these durations being longer in the groups with complications. Furthermore, moderate to strong negative correlations were found between the duration of invasive ventilation (Pearson’s r= −0.56), ICU time (Pearson’s r = −0.61), and hospitalisation time (Pearson’s r= −0.42) and the presence of complications, indicating that these variables are not only statistically significant but also practically relevant in their relationship with complications.

At one month of postoperative follow-up, the echocardiogram revealed significant differences between the groups regarding the residual maximal instantaneous RVOT gradient (*p* < 0.01), which was higher for the group with complications (25.0 [21.0–30.0] mmHg) compared to the group without complications (20.0 [14.6–25.5] mmHg). However, the correlation between the Residual Maximal instantaneous RVOT gradient and complications was weak (Pearson’s r = −0.23), suggesting that although the difference is statistically significant, the practical relevance of this association is limited. Other echocardiographic parameters did not show significant differences between the groups (Table 4).

### Logistic Regression

All variables with a *p*-value <0.05 that showed significant differences between the groups in analysis, as well as those that were related to the variable “complications”, were selected.

Univariate analysis identified multiple parameters associated with the presence of complications within the first 30 postoperative days: hypoplastic pulmonary valve annulus diameter (measured in cm and Z-Score), RVOT surgical technique with RV-PA conduit, increased duration of CPB, increased duration of invasive ventilation, increased ICU time, increased hospitalisation time, and increased residual maximal instantaneous RVOT gradient (Table 5).

To enhance the applicability of the multivariate logistic regression models, we decided to create separate models by categorising the variables into preoperative, perioperative, and postoperative groups. This approach allows for more nuanced analysis of how different types of variables contribute to the risk of complications, given the large number of variables involved.

Multicollinearity between all independent variables included in the logistic regression models was assessed using Tolerance and VIF. All variables had Tolerance >0.1 and VIF <10, indicating no problematic collinearity (Table S1 in Supplementary Material).

Initially, a multivariate logistic regression analysis was performed to identify significant preoperative and perioperative predictors of complications within the first 30 postoperative days (model 1).

Model 1, which included the variable “pulmonary valve annulus (cm)”, and “surgical technique with RV-PA conduit” was statistically significant [ꭓ^2^ = 19.451; df = 2; *p* < 0.01; -2 Log Likelihood = 84.925; R^2^_Negelkerke_ = 0.275].

The Hosmer–Lemeshow test (ꭓ^2^ = 8.966; df = 8; *p* = 0.35) confirmed a good fit of the model to the data.

The results show that the pulmonary valve annulus size is a significant predictor of complications (OR = 0.066; 95%CI = 0.009–0.490; *p* < 0.01), indicating that for each unit decrease in the pulmonary valve annulus size, the risk of complications increases by 34.6%. The surgical technique with RV-PA conduit is a significant predictor of complications (OR = 13.254; 95%CI = 2.106–83.433; *p* < 0.01), indicating that patients who underwent this technique have significantly higher risk of postoperative complications.

The equation describing this relationship is as follows:

$$P\left(Complications\right)=\frac{e^{\left(1.172-2.718\cdot (pulmonary\;valve\;annulus\;\left(cm\right)+2.584\left(RV-PA\;conduit\right))\right)}}{1+e^{\left(1.172-2.718\cdot (pulmonary\;valve\;annulus\;\left(cm\right)+2.584\cdot (RV-PA\;conduit))\right)}}$$

**Figure 1:**
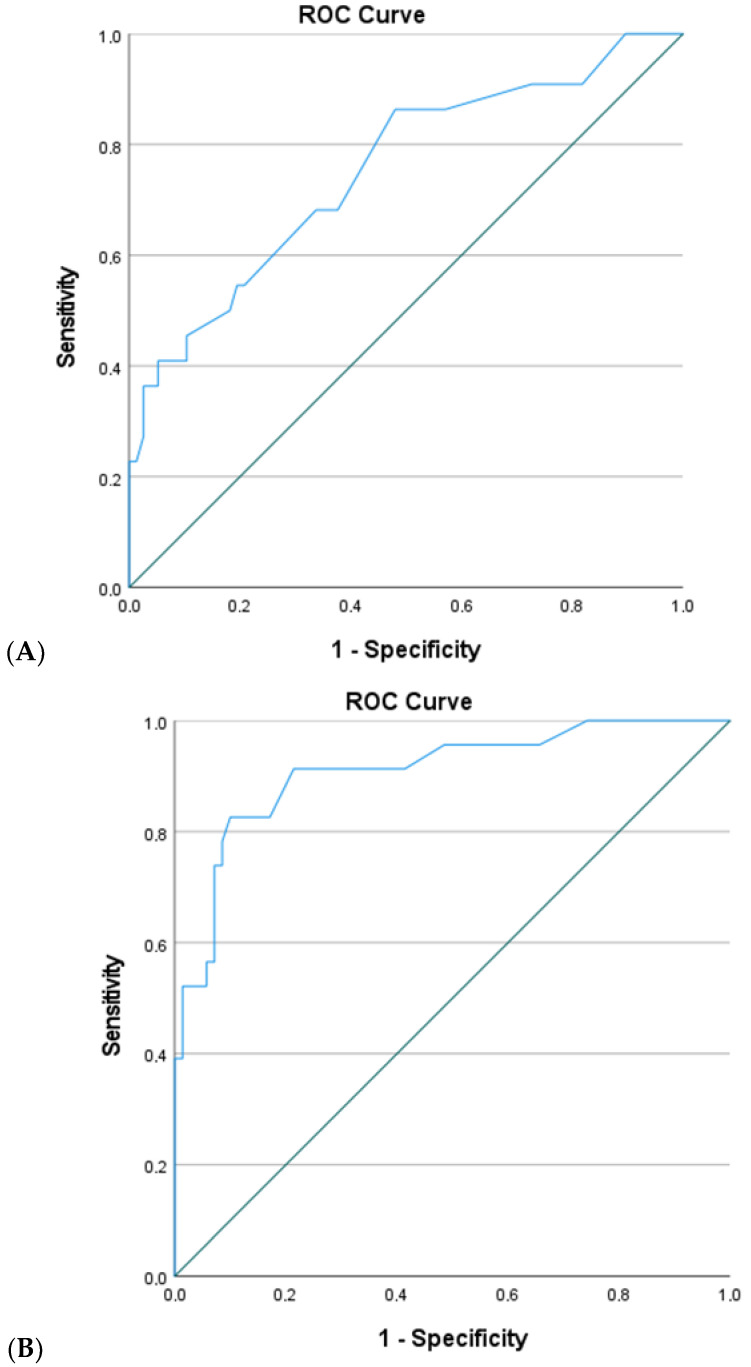
The ROC Curves of the logistic regression model for preoperative and perioperative predictors (**A**) and postoperative predictors (**B**).

The predictive capacity of this equation was assessed using a ROC curve (Figure 1A).

For a sensitivity cutoff of 86.4% and specificity of 51.9%, the area under the curve (AUC) was 0.752 (*p* < 0.01; 95%CI = 0.630–0.874), indicating a moderate association with the presence of complications.

Next, a multivariate logistic regression analysis was performed to identify significant postoperative predictors of complications within the first 30 postoperative days (model 2). Model 2, which included the variable “invasive ventilation time” and “hospitalisation time”, was significant [ꭓ^2^ = 45.024; df = 2; *p* < 0.001; -2 Log Likelihood = 59.018; R^2^_Negelkerke_ = 0.570].

The Hosmer–Lemeshow test (ꭓ^2^ = 14.076; df = 8; *p* = 0.08) suggested an acceptable fit of the model to the data.

The results showed that the “invasive ventilation time” is a significant predictor of complications (OR = 1.068; 95%CI = 1.024–1.115; *p* < 0.01), as well as the “hospitalisation time” (OR = 1.093; 95%CI = 1.003–1.191; *p* = 0.04) indicating that for each additional hour of invasive ventilation time, the risk of complications increase 1.068 times and for each additional day of the hospitalisation time, the risk of complications increase 1.093 times.

The equation describing this relationship is as follows:

$$P\left(Complications\right)=\frac{e^{\left(-3.886+0.066\cdot (invasive\;ventilation\;time\;\left(hours\right)+0.089\cdot (hospitalisation\;time\left(days\right))\right)}}{1+e^{\left(-3.886+0.066\cdot (invasive\;ventilation\;time\;\left(hours\right)+0.089\cdot (hospitalisation\;time\left(days\right))\right)}}$$

The predictive ability of this equation was assessed through the ROC curve analysis (Figure 1B). For a sensitivity cutoff of 91.3% and a specificity of 78.6%, the ROC curve demonstrated an AUC of 0.909 (*p* < 0.01; 95%CI = 0.835–0.983), indicating an excellent capacity to discriminate between patients with and without complications.

## 4. Discussion

Humanitarian paediatric patients undergoing late surgical correction of TOF represent a relatively rare population in developed countries but constitute a significant proportion in developing nations where paediatric cardiac surgery programmes are being established.

In this study, we investigated the risk factors associated with postoperative complications in 115 humanitarian paediatric patients, with a median age of 1444 days (approximately 4 years), who underwent complete surgical correction of TOF.

The data showed that 28 patients (24.3%) experienced complications within the first 30 days post-surgery. Complications included early surgical reintervention in 7% of patients, neurological deficits in 3.5%, and delayed sternal closure in 3.5%. The complication rate observed in our cohort (24.3%) is notable higher than that reported in studies of early TOF repair, which documented a complication rate of 11.6% in patients undergoing surgery at 6 months of age or younger [12]. This difference likely reflects the impact of late repair in patients with more complex clinical conditions, as well as the challenges faced by humanitarian patient population, who often come from developing countries with delayed diagnosis, comorbidities, and limited access to specialised care. These findings highlight the necessity of a thorough evaluation of predictors for these complications to guide clinical management and align with previous studies demonstrating the complexity and challenges of TOF surgical correction, particularly in patients with more severe cardiac and haemodynamic anatomies [8,13].

When analysing preoperative, perioperative and postoperative factors, two multivariate logistic regression models were proposed: Model 1, incorporating pre- and perioperative variables, and Model 2, including postoperative variables.

In Model 1, only variables such as “pulmonary valve annulus diameter (cm)” and the use of “RV-PA conduit” were significantly associated with the occurrence of complications within the first 30 days postoperatively. For each mm decrease in the pulmonary valve annulus, the risk of complication increased by 34.6% (OR = 0.066; 95% CI = 0.009–0.490; *p* < 0.01), while the use of an RV-PA conduit increased the risk by 13-fold (OR = 13.252; 95% CI = 2.106–83.433; *p* < 0.01). Additionally, univariate analysis indicated that PV-SR significantly reduced the risk of complications by 77.5% (OR = 0.225), which is supported by other studies [3,14].

A cohort study comparing long-term outcomes of patients undergoing transannular patch repair versus valve sparing techniques observed that, while the valve sparing technique was beneficial, patients with severe hypoplasia often required RV-PA conduits, which could increase the risk of complications [14]. Pulmonary valve preservation is advocated to achieve the best long-term outcomes and reduce pulmonary regurgitation [3,14].

Another study highlighted that valve sparing technique can reduce the risk of reinterventions and complications, even in the presence of moderate residual stenosis, as they reduce pulmonary regurgitation, preserve right ventricular function, and decrease the incidence of late arrhythmias, which are key determinants of long-term outcomes [8,14].

The choice of surgical technique must consider the severity of pulmonary annulus hypoplasia and the patient’s clinical condition.

Although we did not have access to valve tissue quality evaluation in the surgical report due to the retrospective nature of the study, we believe that chronic remodelling induced by turbulent flow and increased shear stress on the pulmonary valve may compromise the elasticity and functionality of the valve tissue, making preservation of the native valve annulus unfeasible in some cases, leading to the use of an RV-PA conduit. A cohort study also suggested increased complication risks in patients requiring conduits or patches for severe hypoplasia [15].

Using multivariate logistic regression, we developed a probability equation for complications within the first 30 days post-surgery, based on the ROC curve cut-off points. The equation of Model 1 may serve as a clinical decision-making tool, helping to stratify patients by complication risk and implement preventive strategies. By optimising decision-making in this population, healthcare providers may reduce complications and associated direct and indirect costs.

In Model 2, “invasive ventilation time” and “hospitalisation time” were significantly associated with complications within the first 30 days postoperatively. For each additional hour of invasive ventilation, the risk of complications increased by 1.68 times (OR = 1.068; 95% CI = 1.024–1.115; *p* < 0.01), and for each additional day of hospitalisation, the risk increased by 1.093 times (OR = 1.093; 95% CI = 1.003–1.191; *p* = 0.04). These findings are widely supported in the literature, which recognises prolonged invasive ventilation and extended hospitalisation as factors associated with an increased risk of postoperative complications [16].

However, it is important to consider that prolonged invasive ventilation and extended hospitalisation might not only be risk factors but also consequences of postoperative complications. Patients experiencing complications often require longer ventilatory support and hospital stay, which introduces the possibility of reverse causality in this observational study.

Prolonged mechanical ventilation may increase the risk of morbidities such as arrhythmias and infectious complications, which in turn may elevate mortality rates [16]. Extended hospitalisation may indicate haemodynamic or infectious complications, which elevate the risk of reoperations [17]. It should be noted that some variables included in the models, such as prolonged invasive ventilation and extended hospitalisation, could act as potential confounders or lie on the causal pathway. For example, longer ventilation may both reflect and contribute to complications, while prolonged hospital stay may result from or exacerbate morbidities. These interrelationships were carefully considered when interpreting the model results, acknowledging that associations may reflect both direct and indirect effects.

Model 2 highlights the evolution of complications and their effects on hospital outcomes, providing valuable insights for continuous monitoring and adjusting clinical interventions. By identifying the relationship between ventilation and hospitalisation durations with complications, healthcare professionals can take a more proactive approach, reducing these durations, preventing complications, improving resources forecasting, and planning discharges more efficiently.

Thus, while Model 1 focused on pre- and perioperative variables to predict complication risk, Model 2 explores how complications manifest after surgery and how they affect outcomes. This allows for a more comprehensive understanding of the impact of complications and helps refine ongoing postoperative risk assessment and postoperative care.

Although corrective surgery for TOF has advanced significantly, with very low mortality rates, a substantial proportion of patients still face postoperative complications. Our results highlight the importance of individualised management protocols, particularly for humanitarian paediatric patients. Factors such as pulmonary annulus size and surgical techniques, like RV-PA conduit, should be considered during preoperative evaluation. Additionally, close monitoring of invasive ventilation time, and hospitalisation duration may help identify at-risk patients early, allowing for targeted interventions.

This study has some limitations. The analysis was conducted at a single institution, which may limit the generalizability of the results. While the sample size was adequate to identify significant predictors, it may not be sufficient to explore all possible variables. Multicentre studies with larger samples would be beneficial to confirm our findings and expand the understanding of complications associated with TOF correction in humanitarian paediatric patients.

The heterogeneity in the definition and severity of complications might have influenced our results, and the retrospective nature of the study limited access to some echocardiographic data. A more detailed evaluation of echocardiographic parameters of right ventricular function would have been beneficial, as these markers provide important prognostic information post-repair. Due to the retrospective design of the study, systematic genetic evaluation was not available. Although some patients may have had phenotypic features suggestive of genetic syndromes, no formal testing was performed, and clinical suspicion was not consistently documented in the medical records. This represents a limitation, as unrecognised genetic anomalies may have influenced the outcomes.

## 5. Conclusions

This study demonstrated that surgical correction of TOF in humanitarian paediatric patients can be successfully performed, with low morbidity and mortality rates, even in a population with challenging characteristics. PV-SR emerged as an approach that minimises postoperative complications. In contrast, analysis of our Model 1 showed that a hypoplastic pulmonary valve annulus and the implantation of an RV-PA conduit were significant predictors of postoperative complications.

Additionally, as analysed through Model 2, prolonged invasive ventilation and hospitalisation times were significantly associated with an increased risk of postoperative complications, serving as important indirect markers of the severity of these complications.

The use of the proposed predictive models can facilitate proactive patient monitoring by identifying high-risk patients preoperatively and guiding postoperative triaging. This enables clinicians to implement tailored interventions—such as closer monitoring, early therapeutic adjustments, or intensive care resource allocation—thereby improving outcomes and potentially reducing complication-related costs.

## Figures and Tables

**Table 1:** Demographic, physical and laboratory characteristics.

	Total	Complications (<30 Days)		*p*	Effect Size
		No	Yes		
	(n = 115)	(n = 87)	(n = 28)		
Gender ^1^					
Male	69 (60)	54 (62.1)	15 (53.6)	0.51 ^4^	0.07 ^8^
Female	46 (40)	33 (37.9)	13 (46.4)		
Country of origin ^1^					
Algeria	6 (5.2)	3 (3.4)	3 (10.7)	0.20 ^5^	0.43 ^8^
Burkina Faso	1 (0.9)	1 (1.1)	0 (0.0)		
Benin	19 (16.5)	15 (17.2)	4 (14.3)		
Cameroun	1 (0.9)	1 (1.1)	0 (0.0)		
Chad	1 (0.9)	1 (1.1)	0 (0.0)		
Côte d’Ivoire	1 (0.9)	1 (1.1)	0 (0.0)		
Djibouti	1 (0.9)	0 (0.0)	1 (3.6)		
Georgia	1 (0.9)	0 (0.0)	1 (3.6)		
Guinea	12 (10.4)	8 (9.2)	4 (14.3)		
Maroc	15 (13.0)	8 (9.2)	7 (25.0)		
Mali	13 (11.3)	11 (12.6)	2 (7.1)		
Mauritania	15 (13.0)	13 (14.9)	2 (7.1)		
Niger	1 (0.9)	1 (1.1)	0 (0.0)		
Polonia	2 (1.7)	1 (1.1)	1(3.6)		
Senegal	13 (11.3)	10 (11.5)	3 (10.7)		
Syria	1 (0.9)	1 (1.1)	0 (0.0)		
Togo	5 (4.3)	5 (5.7)	0 (0.0)		
Tunisia	7 (6.1)	7 (8.0)	0 (0.0)		
Age at surgical repair (days) ^2^	1444 [942.0–2337.0]	1444 [982.0–2337.0]	1270.5 [698.8–2405.3]	0.40 ^6^	−0.08 ^9^
Weight (Kg) ^2^	12.2 [10.0–16.5]	12.9 [10.0–16.5]	11 [8.0–16.8]	0.12 ^6^	−0.15 ^9^
Height (cm) ^2^	97.0 [85.0–115.0]	99.0 [87.0–115.0]	93.0 [73.5–114.5]	0.13 ^6^	−0.14 ^9^
BMI (Kg/m^2^) ^2^	13.5 [12.5–14.7]	13.4 [12.4–14.7]	13.6 [12.6–14.8]	0.70 ^6^	−0.04 ^9^
Blood Group ^1^					
A+	27 (23.5)	20 (23.0)	7 (25.0)	0.32 ^5^	0.23 ^8^
O+	51 (44.3)	40 (46.0)	11 (39.3)		
B+	23 (20.0)	19 (21.8)	4 (14.3)		
O-	9 (7.8)	5 (5.7)	4 (14.3)		
AB+	4 (3.5)	3 (3.4)	1 (3.6)		
B-	1 (0.9)	0 (0.0)	1 (3.6)		
Peripheral pulse oximetry (%) ^2^	80 [70–87]	82 [71.5–87]	75 [63–85]	0.07 ^6^	−0.17 ^9^
Haemoglobin (g/dL) ^3^	17.2 ± 4.1	17.2 ± 4.1	17.4 ± 4.4	0.81 ^7^	−0.05 ^10^

*Abbreviations*: BMI—Body Mass Index; Kg—kilograms; cm—centimetre; m^2^—metres quarter; %—percent; g—grams; *p*—*p*-value for general statistical tests. ^1^ count (%). ^2^ median [interquartile range]. ^3^ mean ± standard deviation. ^4^ Pearson’s chi-square test. ^5^ Fisher’s exact test. ^6^ Mann–Whitney test. ^7^ Independent samples *t*-test. ^8^ Cramer’s V test. ^9^ Cohen’s d test. ^10^ Pearson’s r (derived from the Z statistic) test.

**Table 2:** Preoperative parameters.

	Total	Complications (<30 Days)		*p*	Effect Size
		No	Yes		
	(n = 115)	(n = 87)	(n = 28)		
**Cardiac Associated malformations**					
Mitral Regurgitation ^1^					
No	107(93.0)	79 (90.8)	28 (100.0)	0.20 ^4^	0.07 ^8^
Yes	8(7.0)	8 (9.2)	0 (0.0)		
Tricuspid Regurgitation ^1^					
No	95 (82.6)	72 (82.8)	23 (82.1)	1.00 ^4^	0.02 ^8^
Yes	20 (17.4)	15 (17.2)	5 (17.9)		
Aberrant Coronary Artery ^1^					
No	109 (95.6)	83 (96.5)	26 (92.9)	0.60 ^4^	0.08 ^8^
Yes	5 (4.4)	3 (3.4)	2 (7.4)		
Interatrial Shunt ^1^					
No	26 (22.6)	20 (23.0)	6 (21.4)	1.00 ^5^	0.02 ^8^
Yes	89 (77.4)	67 (77.0)	22 (78.6)		
Persistent ductus arteriosus ^1^					
No	63 (55.3)	49 (56.3)	14 (51.9)	0.83 ^5^	0.04 ^8^
Yes	51 (44.7)	38 (43.7)	13 (48.1)		
MAPCAs ^1^					
No	90 (86.5)	69 (86.3)	21 (87.5)		
Yes	14 (13.5)	11 (13.8)	3 (12.5)	1.00 ^4^	0.02 ^8^
Anomaly of the systemic venous return ^1^					
No	108 (93.9)	82 (94.3)	26 (92.9)		
Yes	7(6.1)	5 (5.7)	2 (7.1)	0.68 ^4^	0.03 ^8^
Right Aortic Arch ^1^					
No	97 (85.1)	74 (86.0)	23 (82.1)	0.76 ^4^	0.05 ^8^
Yes	17 (14.9)	12 (14.0)	5 (17.9)		
**Preoperative echocardiography**					
Maximal instantaneous RVOT gradient (mmHg) ^2^	75.0 [70.0–83.8]	75.0 [70.0–85.0]	70.0 [70.0–77.5]	0.45 ^6^	−0.08 ^10^
Pulmonary valve annulus (cm) ^3^	1.1 ± 0.3	1.1 ± 0.3	0.9 ± 0.3	<0.01 ^7^	0.81 ^9^
Pulmonary valve annulus Z-Score ^2^	−2.0 [(−2.8)–(−1.1)]	−1.91 [(−2.51)–(−1.16)]	−2.7 [(−4.7)–(−1.0)]	<0.01 ^6^	−0.26 ^10^

*Abbreviations*: *MAPCAs*—Major Aortopulmonary Collateral Arteries; *RVOT*—Right ventricular outflow tract; *mmHg*—millimetres of mercury; *cm*—centimetre; *p*—*p*-value for general statistical tests. ^1^ count (%). ^2^ median [interquartile range]. ^3^ mean ± standard deviation. ^4^ Fisher’s exact test. ^5^ Pearson’s chi-square test. ^6^ Mann–Whitney test. ^7^ Independent samples *t*-test. ^8^ Cramer’s V test. ^9^ Cohen’s d test. ^10^ Pearson’s r (derived from the Z statistic) test.

**Table 3:** Perioperative characteristics.

	Total	Complications (<30 Days)		*p*	Effect Size
		No	Yes		
	(n = 115)	(n = 87)	(n = 28)		
Surgical Urgency ^1^					
Elective	87 (77.0)	68 (80.0)	19 (67.9)	0.20 ^3^	0.13 ^6^
Urgent	26 (23.0)	17 (20.0)	9 (32.1)		
RVOTO Surgical Technique ^1^					
PV-SR	85 (73.9)	71 (81.6)	14 (50.0)	<0.01 ^4^	0.35 ^6^
Transannular patch	17 (14.8)	11 (12.6)	6 (21.4)		
RV-PA conduit	13 (11.3)	5 (5.7)	8 (28.6)		
CPB time (min) ^2^	71.0 [56.0–90.0]	67.0 [54.8–87.0]	87.5 [64.3–96.8]	<0.01 ^5^	−0.23 ^7^
ACC time (min) ^2^	41.0 [30.0–52.0]	39.0 [30.0–50.0]	45.5 [26.0–64.5]	0.67 ^5^	−0.04 ^7^

*Abbreviations*: *RVOTO*—Right ventricular outflow tract obstruction; *PV*-SR—Pulmonary valve-sparing repair; *RV-PA*—Right ventricle to pulmonary artery; *min*—minutes; *CPB*—cardiopulmonary bypass; *ACC*—Aortic Cross Clamping; *p*—*p*-value for general statistical tests. ^1^ count (%). ^2^ median [interquartile range]. ^3^ Pearson’s chi-square test. ^4^ Fisher’s exact test. ^5^ Mann–Whitney test. ^6^ Cramer’s V test. ^7^ Pearson’s r (derived from the Z statistic) test.

**Table 4:** Postoperative outcomes.

	Total	Complications (<30 Days)		*p*	Effect Size
		No	Yes		
	(n = 115)	(n = 87)	(n = 28)		
**Postoperative periods**					
Invasive ventilation time (hours) ^1^	16.0 [8.0–24.0]	16.0 [8.0–24.0]	48.0 [24.0–120.0]	<0.01 ^3^	−0.56 ^6^
ICU time (days) ^1^	3.0 [2.0–3.5]	2.0 [2.0–3.0]	5.0 [3.0–10.5]	<0.01 ^3^	−0.61 ^6^
Hospitalisation time (days) ^1^	6.0 [5.0–8.3]	6.0 [5.0–7.0]	11.5 [6.0–25.8]	<0.01 ^3^	−0.42 ^6^
**Postoperative echocardiography**					
Residual Maximal instantaneous RVOT gradient (mmHg) ^1^	22.0 [15.0–28.0]	20.0 [14.6–25.5]	25.0 [21.0–30.0]	<0.01 ^3^	−0.23 ^6^
Residual VSD ^2^					
No	49 (84.5)	38 (82.6)	11 (91.7)	0.67 ^4^	0.10 ^7^
Yes	9 (15.5)	8 (17.4)	1 (8.3)		
Residual Pulmonary Regurgitation^2^					
No	45 (40.9)	34 (40.0)	11 (44.0)	0.82 ^5^	0.03 ^7^
Yes	65 (59.1)	51 (60.0)	14 (56.0)		
Degree of Residual pulmonary Regurgitation ^2^					
Minimal	22 (34.4)	20 (40.0)	2 (14.3)	0.21 ^4^	0.25 ^7^
Mild	30 (46.9)	22 (44.0)	8 (57.1)		
Moderate	11 (17.2)	7 (14.0)	4 (28.6)		
Severe	1 (1.6)	1 (2.0)	0 (0.0)		

*Abbreviations*: *ICU*—Intensive care unit; *RVOT*—Right ventricular outflow tract; mmHg—millimetres of mercury; *VSD*—Ventricular Septal Defect; *p*—*p*-value for general statistical tests. ^1^ median [interquartile range]. ^2^ count (%). ^3^ Mann–Whitney test. ^4^ Fisher’s exact test. ^5^ Pearson’s chi-square test. ^6^ Pearson’s r (derived from the Z statistic) test. ^7^ Cramer’s V test.

**Table 5:** Univariate regression analysis.

	OR	[95% CI]	*p*
**Preoperative echocardiography**			
Pulmonary valve annulus (cm)	0.053	[0.008–0.374]	<0.01
Pulmonary valve annulus (Z-Score)	0.680	[0.511–0.907]	<0.01
**Perioperative parameters**			
RVOTO Surgical Technique			
PV-SR	0.225	[0.090–0.564]	<0.01
Transannular patch	0.260	[0.626–5.673]	0.26
RV-PA conduit	6.560	[1.938–22.211]	<0.03
CPB time (min)	1.020	[1.004–1.035]	0.01
**Postoperative periods**			
Invasive Ventilation time (hours)	1.068	[1.028–1.110]	<0.01
ICU time	2.240	[1.502–3.339]	<0.01
Hospitalisation time	1.202	[1.094–1.320]	<0.01
**Postoperative echocardiography**			
Residual Maximal instantaneous RVOT gradient (mmHg)	1.057	[1.003–1.113]	0.04

*Abbreviations*: *OR*—Odds Ratio; *95%CI*—95% confidence interval; *RVOTO*—Right ventricular outflow tract obstruction; *PV-SR*—Pulmonary valve- sparing repair; *RV-PA*—Right ventricle to pulmonary artery; *CPB*—Cardiopulmonary bypass; *ICU*—Intensive Care Unit; *RVOT*—Right ventricular outflow tract; min—minutes; mmHg—millimetres of mercury; *p*—*p*-value for general statistical tests.

## Data Availability

The datasets used and/or analysed during the cur-rent study are available from the corresponding author on reasonable request.

## Supplementary Materials

The following supporting information can be downloaded at: https://www.mdpi.com/article/10.3390/children12091111/s1, Table S1: Multicollinearity assessment of independent variables included in logistic regression models.

## Abbreviations

TOF	Tetralogy of Fallot
VSD	Ventricular Septal Defect
RVOT	Right Ventricular Outflow Tract
CPB	Cardiopulmonary Bypass
RVOTO	Right Ventricular Outflow Tract Obstruction
PV-SR	Pulmonary Valve-Sparing Repair
RV-PA Conduit	Right Ventricle to Pulmonary Artery Conduit
BMI	Body Mass Index
ICU	Intensive Care Unit
ECMO	Extracorporeal Membrane Oxygenation
SPSS	Statistical Package for the Social Sciences
SW	Shapiro–Wilk test
OR	Odds Ratio
95% CI	95% Confidence Interval
VIF	Variance Inflation Factor
ROC Curve	Receiver Operating Characteristics Curve
MAPCAs	Major Aortopulmonary Collateral Arteries
AUC	Area Under the Curve
