# Supplementary material for: Postoperative Complications in Humanitarian Paediatric Patients Undergoing Late Surgical Correction of Tetralogy of Fallot: A Multivariate Analysis
Source: Children (Basel). 2025 Aug 23;12(9):1111. doi: 10.3390/children12091111 (PMC12468921; doi:10.3390/children12091111)
Supplement: Supplementary file 1 [file children-12-01111-s001.zip › children-3817378-supplementary.pdf]

Table S1: Multicollinearity assessment of independent variables included in logistic regression models.

	Tolerance	VIF
**Preoperative echocardiography**		
Pulmonary valve annulus (cm)	0.72	1.39
Pulmonary valve annulus (Z-Score)	0.66	1.52
**Perioperative parameters**		
RVOTO Surgical Technique	0.45	2.21
CPB time (min)	0.71	1.42
**Postoperative periods**		
Invasive Ventilation time (hours)	0.34	2.96
ICU time	0.31	3.25
Hospitalisation time	0.44	2.27
**Postoperative echocardiography**		
Residual Maximal instantaneous RVOT gradient (mmHg)	0.89	1.12

Abbreviations: VIF-Variance Inflation Factor; *RVOTO*-Right ventricular outflow tract obstruction; *CPB*-Cardiopulmonary bypass; *ICU*-Intensive Care Unit; *RVOT*-Right ventricular outflow tract; cm-centimetre; min-minutes; mmHg-millimetres of mercury.
